# Long-term prediction of multidimensional social inclusion among patients with schizophrenia spectrum disorder

**DOI:** 10.1192/j.eurpsy.2023.651

**Published:** 2023-07-19

**Authors:** J. Hao, N. Tiles-Sar, L. van der Meer, R. Bruggeman, B. Z. Alizadeh

**Affiliations:** ^1^Department of Epidemiology, University Medical Center Groningen; ^2^Department of Clinical and Developmental Neuropsychology, Faculty of Behavioural and Social Sciences, University of Groningen, Groningen; ^3^Department of Rehabilitation, Lentis Psychiatric Institute, Zuidlaren; ^4^Department of Psychiatry, University Medical Center Groningen, Groningen, Netherlands

## Abstract

**Introduction:**

Poor social inclusion, as a cause and consequence simultaneously, has been associated with schizophrenia spectrum disorder (SSD). It can bring a substantial burden to individual families and the society. Previous studies lack 1) the quantitative exploration of (multidimensional) social inclusion which can enable the measurement and monitor of the level of social integration, 2) longitudinal and multivariate study designs, and 3) methodological comparison between the traditional and data-driven approaches for a better clinical suitability of monitoring and managing social inclusion.

**Objectives:**

To build and compare 3-year models predictive of multidimensional social inclusion (mSI) among the SSD patients, using standard and data-driven approaches.

**Methods:**

We used the baseline and 3-year follow-up data of 1,119 patients from the Genetic Risk and Outcome in Psychosis. Social functioning (Social Functioning Scale, SFS) and quality of life (the brief version of the World Health Organization Quality of Life, WHOQOL-BREF) were used as a proxy of mSI. K-means clustering over the 13 subscales of SFS and WHOQOL-BREF was applied to identify mSI subgroups. Prediction models were built and internally validated via multinomial logistic regression (MLR) and random forest (RF). The MLR and RF model performance was compared by accuracy and the discriminability of mSI subgroups (i.e., p-value of one-sided binomial test between the accuracy and no information rate).

**Results:**

Five mSI groups were identified: 1) “very low (in SFS)/very low (in WHOQOL-BREF)” (8.58%), 2) “low/low” (12.87%), 3) “high/low” (49.24%), 4) “medium/high” (18.05%), and 5) “high/high” (11.26%). Both MLR and RF models included 22 predictors and demonstrated accuracies of 59.16% (95CI%: [55.75%, 62.58%], p = 0.994) and 61.61% (95%CI: [54.90%, 68.01%], p = 0.013) correspondingly. The mSI was robustly and mainly and robustly predicted by genetic predisposition, premorbid social functioning, symptoms (i.e., positive, negative and depressive), number of met needs and baseline satisfaction with the environment and social life.

**Image:**

**Figure:**
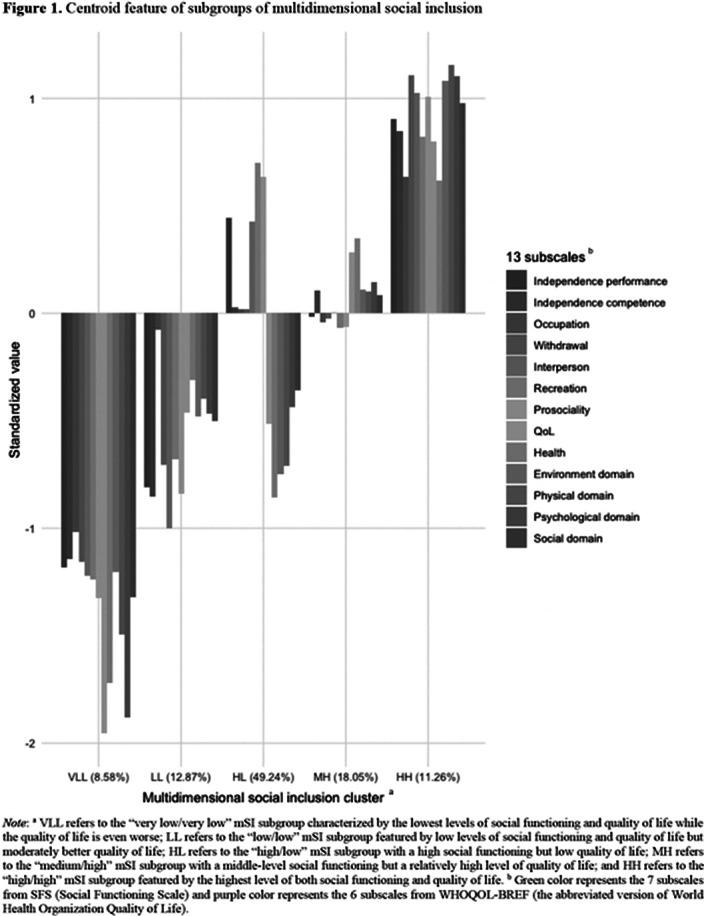

**Image 2:**

**Figure:**
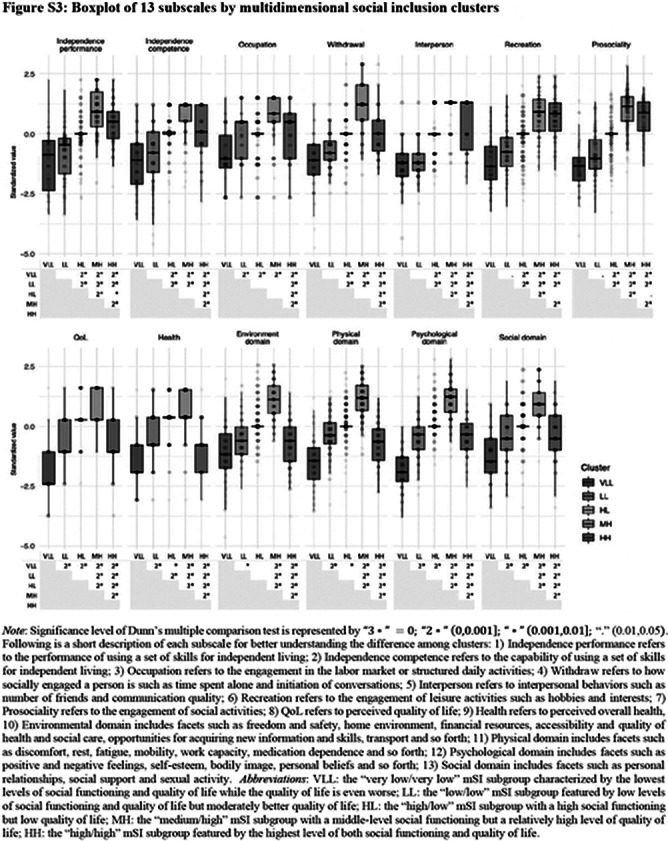

**Image 3:**

**Figure:**
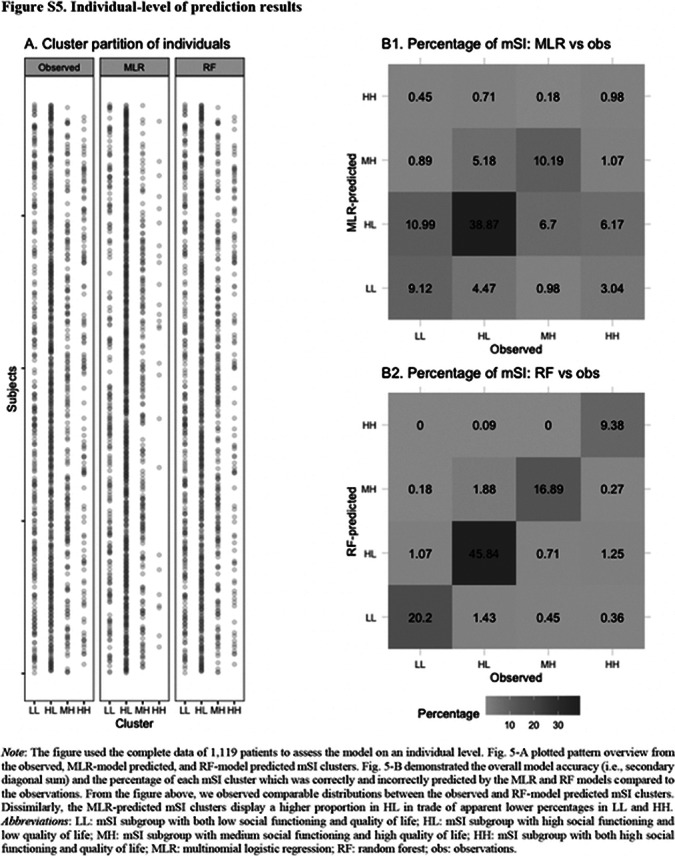

**Conclusions:**

Notwithstanding comparable accuracies, we cautiously consider the RF model outperforming primarily due to its better discriminability. As the baseline conditions of the patients with SSD could indicate the 3-year mSI level, customized amount and types of resources and interventions can be designed to improve the level of multidimensional social inclusion of all SSD patients.

**Disclosure of Interest:**

None Declared

